# Evaluation of a Digital Intervention to Improve the Health Outcomes of Older Adults: Secondary Data Analysis

**DOI:** 10.2196/62748

**Published:** 2026-06-05

**Authors:** Jowinn Chew, Georgina Bartlett, Lily Hopkins, Dan Frings

**Affiliations:** 1 Young Epilepsy, St Piers Lane, Lingfield, RH7 6PW, United Kingdom, 44 01342 832243; 2 School of Allied Health and Life Sciences, College of Health and Life Sciences, London South Bank University, 103 Borough Road, London, SE1 0AA, United Kingdom, 44 020 7815 7815; 3 London School of Hygiene and Tropical Medicine, Faculty of Epidemiology and Population Health, London, United Kingdom

**Keywords:** mobile health strategies, mHealth strategies, mobile health, mHealth, digital health, healthy aging, health outcomes, health behaviors

## Abstract

**Background:**

The health of aging populations is among the top challenges facing global health systems. The use of mobile health (mHealth) approaches has been found to be effective in prompting changes and has been identified as a potentially valuable tool to enhance health behaviors among older adults.

**Objective:**

This study sought to explore the efficacy and acceptability of a personalized mHealth app for older adults, focusing on well-being, mental and physical health, and relationship with food.

**Methods:**

This secondary data analysis examined outcomes of real-world Holly Health users who enrolled between August 2022 and January 2023.

**Results:**

Results showed that, after the intervention, self-confidence, energy, mindfulness, health mindset, and short- and long-term mindset all improved. Furthermore, the personalized mHealth app showed a good level of acceptability among the participants.

**Conclusions:**

Engaging with this digital health intervention improved several aspects of physical and mental health, adding to existing evidence that effective and accessible tools are needed to promote healthy aging.

## Introduction

The global aging population is increasing, with the World Health Organization predicting that the population aged over 60 years will double between 2015 and 2050 to an estimated 2.1 billion [1]. However, research suggests that increased life expectancy does not result in people having more years in “good health” [2]. Instead, as we age, we tend to experience a decline in mobility and mental health, along with an increase in coexisting health conditions such as cardiovascular diseases and other noncommunicable diseases (NCDs) [2-4]. “Healthy aging” refers to the ongoing development and maintenance of functional capabilities that promote well-being in older age [2] and encompasses positive health behaviors such as physical activity [5], eating healthily [6], and not smoking or having a moderate alcohol intake [7], which can reduce the incidence of NCDs when performed throughout the life span. Such behaviors also positively impact well-being in older age [8-10]. Given these benefits and the fact that NCDs are responsible for a large portion of morbidity, mortality, and health care expenditures in older age [11], more research is needed to determine how to promote positive health behaviors in an aging population.

Due to the rise in mobile phone and internet use, mobile health (mHealth) approaches are increasingly being used to deliver interventions [12]. They have been found to be effective in prompting changes across a range of health behaviors and in populations with a range of NCDs [13-15]. While much research has investigated mHealth interventions for younger age groups, there is evidence that they may also be a useful tool for older adults. Mobile phone ownership among this group is growing, with an estimated 78% of those aged over 55 years in the United Kingdom owning a smartphone [16]. A scoping review of mHealth interventions for adults aged over 60 years reported improvements in physical activity, chronic disease management, and medication adherence across studies, suggesting that this intervention type can be effective for older age groups [17]. However, effect sizes for the observed behavior changes tend to be small [18]. One way to maximize effectiveness is through personalization, whereby the intervention is dictated by the choices of the individual [19,20]. Personalized mHealth interventions are largely acceptable [21-23] and more effective for prompting behavior change (eg, increasing physical activity and reducing smoking) compared to nonpersonalized interventions [20,24,25]. This study aimed to evaluate a personalized mHealth app for older adults called Holly Health.

Holly Health targets 4 main health behaviors and constructs for older people (sleep, mental health, exercise, and relationship with food) through personalized features and content. The app incorporates elements of mindfulness therapy, cognitive behavioral therapy (CBT), and acceptance and commitment therapy (ACT). The app includes several features grounded in cognitive behavioral principles. First, the reflective chatbot provides cognitive restructuring prompts, encouraging users to identify unhelpful automatic thoughts (eg, all-or-nothing beliefs about health behaviors) and replace them with more adaptive alternatives. Second, behavioral activation techniques are embedded through graded habit recommendations and small, achievable goals designed to increase engagement in positively reinforcing activities. Third, self-monitoring is facilitated through check-ins and progress tracking, enabling users to notice patterns, triggers, and improvements over time. Fourth, psychoeducational content targets common maladaptive cognitions regarding food, exercise, or self-efficacy, supporting users in challenging rigid beliefs that can act as barriers to change. Finally, the structured goal-setting and problem-solving prompts mirror standard CBT frameworks by helping users break down difficulties, anticipate barriers, and plan coping responses. Features of the app include personalized goal setting, progress tracking (termed “self-monitoring”), habit recommendations, personalized nudges and reminders, and video content and exercises, all aimed at improving attitudes toward health behavior to promote behavior change. The app tailors these features for each user and continuously updates content based on their use of the app. This study aimed to explore the efficacy and acceptability of the Holly Health app for use in older adults, measuring changes in personal well-being, mental and physical health, and relationship with food and thoughts and opinions about the app

## Methods

### Ethical Considerations

This analysis was approved by the London South Bank University Research Ethics Committee (reference ETH2223-0097) and was preregistered on the Open Science Framework and updated on July 11, 2023. All participants provided informed consent, and data were anonymized prior to analysis. After completing the 12-week follow-up, participants were offered a £50 (US $68) voucher of their choice as thanks for taking part.

### Recruitment

Participants were recruited via newsletters, in-person outreach, and posters distributed in local community centers, services, and charities in Lewisham and Southwark, as well as through social media advertisements. Interested individuals were directed to a study landing page and invited to complete a screening questionnaire. Eligibility criteria were age of 50 years or above, fluency in English, an email address, access to a phone or tablet with internet connectivity, and motivation to increase mobility or sustainably manage their weight. Individuals were excluded if they reported a major psychiatric disorder requiring treatment, major physical impairments, or visual impairments not correctable with contact lenses or glasses.

Eligible individuals were sent an information sheet and were asked to complete an onboarding questionnaire assessing current health and well-being habits to tailor program content. As this was a pilot study, no formal sample size calculation was conducted, and recruitment continued until a target sample of approximately 30 participants was reached. Participants enrolled on the Holly Health app between August 2022 and January 2023.

### Intervention

Holly Health is a smartphone app that offers daily health and well-being coaching. It aims to help users prioritize, achieve, and sustain daily health habits across several domains, including exercise, sleep, mental health, and their relationship with food. The app incorporates elements of mindfulness therapy, CBT, and ACT. Features of the app include personalized habit recommendations; a chatbot for reflection exercises and tailored coaching; motivational nudges and reminders; and tailored content in the form of videos, articles, and exercises. The app and its recommendations (including habit reminders, articles, challenges, and reflective exercises) are tailored to each person, and the personalization adapts throughout the time the users engage with the service. Users were asked to use the app 3 to 4 times a week and were prompted to complete measures online at baseline and 8 and 12 weeks.

### Outcome Measures

#### Overview

The schedule of measures can be found in Table 1.

**Table 1.** Schedule of measures.

Outcome measure	Study stage		
	Baseline	Week 8	Week 12
ONS-4^a^	✓	✓	✓
FCQ^b^	✓	✓	
Health survey	✓	✓	✓
Retrospective questions	✓	✓	✓

a ONS-4: Office of National Statistics personal well-being questions.

b FCQ: Food Choice Questionnaire.

#### Office of National Statistics Personal Well-Being Questions

The Office of National Statistics personal well-being questions (ONS-4) measure subjective well-being. This measure consists of 4 scales developed to assess life satisfaction, worthwhileness, happiness, and anxiety. Each item is rated from 0 (“not at all”) to 10 (“completely”). The Cronbach α was 0.83 in this study.

#### Food Choice Questionnaire

The Food Choice Questionnaire (FCQ) comprises 36 items that are designed to assess the health and nonhealth motives influencing an individual’s food choices. This measure consists of 9 scales developed to assess health, mood, convenience, sensory appeal, natural content, price, weight control, familiarity, and ethical concerns as factors related to food choice.

#### Health Survey

The health survey is a tailor-made questionnaire developed by Holly Health and comprises 8 scales aimed at measuring different aspects of an individual’s health: physical activity, self-confidence, relationship with food, energy levels, mindfulness, short- vs long-term mindset, health mindset, and self-kindness. This measure is not designed to provide an aggregate score but, rather, to illustrate different aspects of an individual’s health. This questionnaire is provided in Multimedia Appendix 1.

#### Retrospective Use and User Experience Questions

At weeks 8 and 12, users were asked 4 questions regarding how often they used the Holly Health app and how useful they found it. These were summarized descriptively. At week 12, users were asked 2 questions about the acceptability of the app. These questions are provided in Multimedia Appendix 2.

### Statistical Analysis

All analyses were exploratory. Formal hypothesis testing was restricted to primary outcomes (health survey self-confidence, energy, mindfulness, short- and long-term mindset, health mindset, and self-kindness subscales; FCQ; and ONS-4); other comparisons were treated as descriptive. Changes from baseline were assessed using paired *t* tests (2-tailed) for continuous variables and Wilcoxon signed-rank tests for ordinal or categorical domains. Effect sizes (Cohen *d* for *t* tests; *r* or *d* for the Wilcoxon signed-rank test) and 95% CIs were reported where available. Regression models examined baseline predictors of outcomes, and sensitivity analyses assessed moderation by app use frequency. Given the small sample size (N=34), analyses were underpowered, and the findings should be interpreted cautiously.

To evaluate the efficacy of the app in improving people’s attitudes toward their health and food choices, changes in scores on the health survey and FCQ compared to baseline were assessed using repeated-measure *t* tests for all continuous data and the Wilcoxon signed-rank test of differences for all categorical data. Subscales 1 (physical activity), 2 (self-confidence), 4 (energy), 5 (mindfulness), 7 (health mindset), and 8 (self-kindness) on the health survey and the FCQ were coded as continuous data. Subscales 3 (relationship with food) and 6 (short- and long-term mindset) on the health survey were coded as categorical data. To assess whether users’ attitudes toward their health and food choices predicted life satisfaction, logistical regressions on the ONS-4 were conducted at 8 and 12 weeks, predicted by scores on the health survey and FCQ (collected at baseline). A sensitivity analysis was undertaken on all continuous data by analyzing the number of times the app was used over the course of the trial as a moderator variable. This was achieved using the PROCESS macro by Hayes [26]. The sensitivity analysis was not conducted on categorical data. Finally, participants’ responses to the retrospective questions (whether they reported using the app at least once a week and whether they reported that they had started to practice any of the habits automatically) were used to assess the acceptability of the app.

## Results

### Baseline Demographics

**Figure 1.**
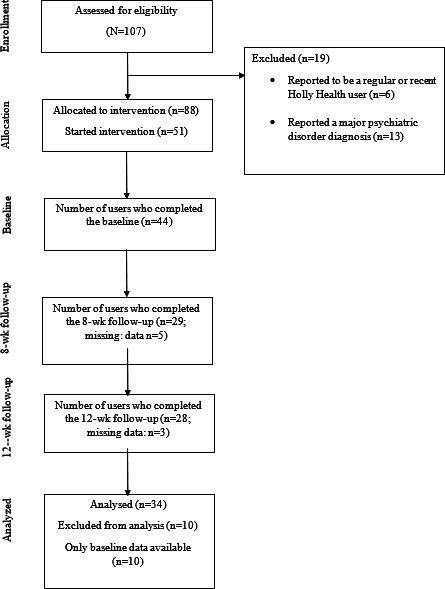
ou maStudy flowchart.

Participant flow is shown in Figure 1. Most users were women (28/36, 77.7%), aged between 50 and 59 years (18/36, 50%), and White (25/36, 69.4%) and reported having hypertension (11/36, 30.6%). Table 2 presents the sample demographics.

**Table 2.** User characteristics at baseline.

Variable	Participants (N=36)^a^, n (%)
Gender	
Woman	28 (77.7)
Man	8 (22.3)
Age range (y)	
50-59	18 (50)
60-65	6 (16.7)
66-70	6 (16.7)
70-75	6 (16.7)
Race or ethnicity	
Asian British	3 (8.3)
Black, African, Caribbean, or Black British	5 (13.9)
Mixed or multiple ethnic groups	1 (2.8)
White	25 (69.4)
Other	1 (2.8)
Not disclosed	1 (2.8)
Medical condition	
Hypertension	11 (30.6)
Arthritis	10 (27.8)
Asthma	4 (11.1)
High cholesterol	11 (30.6)
Type 2 diabetes	6 (16.7)
Heart disease	1 (2.8)
Stroke	1 (2.8)
COPD^b^	1 (2.8)
Osteoporosis	1 (2.8)
Fibromyalgia	1 (2.8)
Cancer	1 (2.8)
Anxiety	10 (27.8)
Depression	7 (19.4)
Other	6 (16.7)
None reported	7 (22.2)

a Demographic characteristics are reported for the 36 participants who completed the baseline assessment and engaged with the intervention period. However, the final longitudinal analysis was conducted on 34 participants. Two participants were excluded from the final comparative analysis because, although they provided partial interval data, they did not meet the full data requirements for the cross-comparison across all primary time points (baseline to week 8/12).

b COPD: chronic obstructive pulmonary disease.

### Evaluation Outcomes

#### Health Survey

Paired-sample *t* tests with Bonferroni correction showed that self-confidence scores were significantly higher at 12 weeks (mean 3.77, SD 0.77) than at baseline (mean 3.31, SD 1.09; *P*=.02; *d*=0.55). Energy scores were significantly higher at 12 weeks (mean 3.46, SD 1.03) than at baseline (mean 2.85, SD 1.12; *P*<.001; *d*=0.67). For mindfulness, scores at 8 weeks (mean 3.39, SD 1.20) were significantly higher than at baseline (mean 3.00, SD 1.06; *P*=.05; *d*=0.32). A Wilcoxon signed-rank test showed that short- vs long-term mindset scores were significantly higher at 8 weeks (mean 3.14, SD 1.22) than at baseline (mean 2.65, SD 1.25; *z*=–2.03; *P*=.04; *d*=0.38) and significantly higher at 12 weeks (mean 3.13, SD 1.28) than at baseline (*z*=–2.45; *P*=.01; *d*=0.44). Table 3 presents these findings.

**Table 3.** Health survey (baseline, week 8, and week 12): paired comparisons.

Subscale	Time point	Participants, n	MD^a^	*t* test (*df; 95% CI*)	*z* score	*P* value	Effect size^b^
Physical activity	Baseline to 8 wk	29	−0.231	−1.223 (28; −0.616 to 0.154)	—^c^	.41	0.227
Physical activity	Baseline to 12 wk	31	−0.154	−1.606 (30; −0.619 to 0.312)	—	>.99	0.288
Physical activity	8 wk to 12 wk	26	0.077	0.811 (25; −0.166 to 0.320)	—	>.99	0.159
Self-confidence	Baseline to 8 wk	29	−0.269	−1.978 (28; −0.661 to 0.122)	—	.27	0.367
Self-confidence	Baseline to 12 wk	31	−0.462^d^	−3.053 (30; −0.870 to −0.053)	—	.02^d^	0.55
Self-confidence	8 wk to 12 wk	26	−0.192	−1.309 (25; −0.569 to 0.185)	—	.61	0.257
Energy	Baseline to 8 wk	29	−0.462	−3.266 (28; −0.787 to −0.136)	—	<.001	0.606
Energy	Baseline to 12 wk	31	−0.615^e^	−3.737 (30; −0.994 to −0.237)	—	<.001^e^	0.67
Energy	8 wk to 12 wk	26	−0.154	−1.162 (25; −0.493 to 0.186)	—	.77	0.228
Mindfulness	Baseline to 8 wk	29	−0.385^d^	−3.041 (28; −0.763 to −0.006)	—	.05^d^	0.32
Mindfulness	Baseline to 12 wk	31	−0.423	−1.827 (30; −0.995 to 0.149)	—	.21	0.328
Mindfulness	8 wk to 12 wk	26	−0.038	−0.171 (25; −0.149 to 0.149)	—	.21	0.033
Short- vs long-term mindset	Baseline to 8 wk	29	−0.491	— (−0.801 to −0.026)	–2.03	.04^d^	0.38
Short- vs long-term mindset	Baseline to 12 wk	31	−0.480	— (−1.042 to −0.119)	–2.45	.01^e^	0.44
Short- vs long-term mindset	8 wk to 12 wk	26	−0.01	— (−0.331 to 0.408)	0.00	.80	0.000
Self-kindness	Baseline to 8 wk	29	−0.385	−1.672 (28; −0.955 to 0.186)	—	.29	0.310
Self-kindness	Baseline to 12 wk	31	−0.154	−1.488 (30; −0.597 to 0.289)	—	>.99	0.267
Self-kindness	8 wk to 12 wk	26	0.231	1.443 (25; −0.179 to 0.641)	—	.48	0.283

a MD: mean difference.

b Cohen *d* for paired *t *tests and *r* for Wilcoxon signed-rank tests.

c Not applicable.

d *P*<.05.

e *P*<.01.

#### FCQ Results

Paired-sample *t* tests were conducted to compare FCQ scores between baseline and 8 weeks. A significant increase was found on the mood subscale, with higher scores at 8 weeks (mean 2.60, SD 0.78) than at baseline (mean 2.16, SD 0.60; *t*_26_=–5.03; *P*<.001; *d*=–0.998). No other subscales showed significant changes. Table 4 presents these findings.

**Table 4.** Food Choice Questionnaire (baseline vs week 8): paired-sample *t* tests.

Subscale	Participants, n	MD^a^	*t* test (*df*; 95% CI)	*P* value	Effect size (Cohen *d*)
Health	27	−0.048	−8.19 (26; −0.169 to 0.073)	.42	−0.163
Mood	27	−0.441^b^	−0.503^b^ (26; −0.621 to –0.261)	<.001^b^	−0.998
Convenience	27	0.022	0.178 (26; −0.235 to 0.279)	.86	0.034
Sensory appeal	27	−0.10	−1.335 (26; −0.254 to 0.054)	.19	−0.282
Natural content	27	0.052	0.485 (26; −0.168 to 0.272)	.63	0.089
Price	27	0.026	0.268 (26; −0.173 to 0.225)	.79	0.050
Weight control	27	−0.015	−0.125 (26; −0.259 to 0.229)	.90	−0.040
Familiarity	27	0.004	0.027 (26; −0.282 to 0.290)	.98	0.000
Ethical concern	27	−0.015	−0.115 (26; −0.278 to 0.249)	.91	−0.037

a MD: mean difference.

b *P*<.01.

### Regression Analyses

Hierarchical regressions were used to investigate which factors (scores on the health survey and FCQ at baseline) predicted scores on each of the ONS-4 scales (life satisfaction, worthwhileness, happiness, and anxiety) at 8 and 12 weeks. Scores on the health survey at baseline were found to be a significant predictor of life satisfaction, worthwhileness, and happiness scores on the ONS-4 at 8 weeks, accounting for 65%, 66%, and 60% of the variance, respectively. The regression coefficients are shown in Table 5. Similarly, scores on the health survey at baseline were also revealed as significant predictorof scores on the ONS-4 worthwhileness scale at 12 weeks, accounting for 51% of the variance. The regression models are shown in Table 6.

Regression analysis revealed that scores on the health survey and FCQ at baseline significantly predicted scores on the ONS-4 happiness scale at 12 weeks, accounting for 84% of the variance. Additionally, both scores on the health survey at baseline alone and scores on the health survey and FCQ at baseline combined were predictive of scores on the ONS-4 anxiety subscale at 12 weeks, accounting for 58% and 83% of the variance, respectively. These regression models are presented in Table 6.

**Table 5.** Hierarchical multiple regression coefficients of Office of National Statistics personal well-being scales (ONS-4) as a function of scores on the health survey and Food Choice Questionnaire (FCQ) at baseline. β is the standardized regression coefficient from final hierarchical model (step 2). Step 1 included baseline health survey variables; step 2 added FCQ subscales.

Predictor			At 8 weeks		At 12 weeks	
			β	*P* value	β	*P* value
ONS-4 overall life satisfaction						
	Health survey					
		Physical activity	−0.02	.95	−0.32	.24
		Self-confidence	0.31	.26	0.36	.23
		Relationship with food	−0.41	.13	−0.22	.44
		Energy	0.74	.03	0.34	.24
		Mindfulness	0.27	.32	0.01	.97
		Short- versus long-term mindset	0.16	.58	−0.01	.98
		Health mindset	−0.36	.34	0.14	.67
		Self-kindness	−0.26	.25	0.12	.62
	FCQ					
		Health	0.28	.46	0.45	.15
		Mood	0.04	.89	−0.50	.13
		Convenience	0.35	.14	0.59	.02^a^
		Sensory appeal	0.09	.78	0.16	.59
		Natural content	0.14	.70	0.00	>.99
		Price	−0.09	.78	−0.36	.17
		Weight control	−0.28	.29	−0.04	.85
		Familiarity	−0.30	.32	−0.28	.30
		Ethical concern	−0.25	.32	−0.34	.14
ONS-4 overall worthwhileness						
	Health survey					
		Physical activity	0.01	.95	−0.15	.61
		Self-confidence	0.51	.03^a^	0.26	.42
		Relationship with food	−0.22	.25	−0.11	.73
		Energy	0.63	.01^a^	0.45	.17
		Mindfulness	−0.01	.96	0.07	.84
		Short- versus long-term mindset	−0.13	.57	−0.24	.47
		Health mindset	0.02	.93	0.25	.50
		Self-kindness	−0.10	.58	0.11	.67
	FCQ					
		Health	0.16	.72	0.24	.46
		Mood	0.10	.77	−0.21	.54
		Convenience	0.18	.52	0.16	.48
		Sensory appeal	−0.08	.84	0.07	.81
		Natural content	0.12	.79	0.25	.39
		Price	0.04	.93	−0.16	.57
		Weight control	−0.14	.65	0.09	.70
		Familiarity	−0.05	.90	−0.31	.29
		Ethical concern	−0.30	.33	−0.40	.12
ONS-4 overall happiness						
	Health survey					
		Physical activity	−0.13	.65	−0.62	.02
		Self-confidence	0.15	.64	−0.01	.97
		Relationship with food	−0.37	.24	−0.29	.25
		Energy	0.97	.0^a^2	0.43	.10
		Mindfulness	0.04	.91	0.24	.39
		Short- versus long-term mindset	0.09	.80	0.12	.65
		Health mindset	−0.15	.73	0.23	.44
		Self-kindness	−0.11	.67	−0.17	.41
	FCQ					
		Health	0.08	.87	0.74	.01
		Mood	0.04	.90	−0.26	.34
		Convenience	0.28	.31	0.44	.03
		Sensory appeal	0.19	.61	0.28	.27
		Natural content	0.28	.53	−0.17	.47
		Price	0.19	.62	−0.18	.41
		Weight control	−0.36	.26	−0.21	.27
		Familiarity	−0.22	.53	−0.10	.67
		Ethical concern	−0.16	.58	−0.63	.007
ONS-4 anxiety						
	Health survey					
		Physical activity	−0.26	.50	0.62	.02
		Self-confidence	−0.46	.28	−0.11	.67
		Relationship with food	0.03	.95	−0.36	.17
		Energy	−0.39	.39	−0.05	.85
		Mindfulness	−0.26	.54	−0.40	.17
		Short- versus long-term mindset	0.15	.73	−0.14	.61
		Health mindset	0.66	.27	0.01	.98
		Self-kindness	0.20	.57	0.11	.60
	FCQ					
		Health	−0.18	.76	−0.09	.73
		Mood	−0.39	.38	0.03	.90
		Convenience	0.04	.91	0.26	.18
		Sensory appeal	0.68	.18	−0.06	.82
		Natural content	−0.16	.78	−0.20	.41
		Price	−0.03	.95	−0.38	.11
		Weight control	−0.16	.69	0.35	.09
		Familiarity	−0.26	.58	−0.26	.29
		Ethical concern	0.00	>.99	−0.34	.11

a *P*<.05.

**Table 6.** Model fit and change statistics at 8 and 12 weeks for the hierarchical multiple regression analysis of Office of National Statistics personal well-being scales (ONS-4).

Predictor and time point		*R* ^2^	Adjusted *R*^2^	Δ*R*^2^	*F* change	*P* value
ONS-4 overall life satisfaction						
	8 weeks	0.833	0.650	0.188	1.12	.43
	12 weeks	0.781	0.457	0.324	1.81	.18
ONS-4 overall worthwhileness						
	8 weeks	0.746	0.266	0.088	0.35	.94
	12 weeks	0.740	0.512	0.228	1.09	.45
ONS-4 overall happiness						
	8 weeks	0.762	0.311	0.163	0.68	.71
	12 weeks	0.835	0.579	0.463	3.42	.03
ONS-4 anxiety						
	8 weeks	0.595	−0.169	0.222	0.55	.81
	12 weeks	0.825	0.554	0.247	1.72	.20

### Sensitivity Analysis

A series of moderated multiple regressions were conducted to predict scores on all outcome measures at 12 weeks from baseline (or 8 weeks for the FCQ), with the number of times the app was used serving as a moderator variable. No interaction terms were statistically significant in the models.

### Responses to the Retrospective Use and User Experience Questions

Most individuals used the Holly Health app at least once per week at both 8 and 12 weeks (27/34 79.4% and 30/34, 88.2%, respectively). Of those who reported using the Holly Health app at least once per week, most reported that they had started to practice some of the habits automatically without relying on the app to remind them at both 8 and 12 weeks (24/34, 70.6% and 28/34, 82.4%, respectively). Most respondents indicated that they found the Holly Health app useful at both 8 and 12 weeks (26/34, 76.5% and 27/34, 79.4%, respectively). At 8 weeks, most users (29/34, 85.3%) stated that they would like their local council to provide more services such as this one in the future. Finally, at 12 weeks, most users (47.1%) stated that they found the app to be a very appropriate tool for older adults to keep up with their healthy aging goals and that it would be extremely likely for them to recommend the app to other people with similar health and well-being goals to their own. Table 7 presents these findings.

**Table 7.** Responses to retrospective questions.

Question and time point	Yes, n (%)	No, n (%)	No response, n (%)
Have you been using the Holly Health app at least once a week?			
Week 8	27 (79.4)	2 (5.9)	5 (14.7)
Week 12	30 (88.2)	1 (2.9)	3 (8.8)
Have you started to do any of your habits automatically?			
Week 8	24 (70.6)	3 (8.8)	7 (20.6)
Week 12	28 (82.4)	2 (5.9)	4 (11.8)
Overall, have you found Holly Health useful?			
Week 8	26 (76.5)	1 (2.9)	7 (20.6)
Week 12	27 (79.4)	3 (8.8)	4 (11.8)
In the future, would you like to see more services like this provided by your local council?			
Week 8	29 (85.3)	0 (0)	5 (14.7)

## Discussion

This study presents a secondary data analysis of the Holly Health app. There were significant positive outcomes on the health survey over the trial duration. Specifically, the self-confidence, energy, mindfulness, health mindset, and short- and long-term mindset subscale scores were all higher after the intervention. Participants also rated mood as significantly more important on the FCQ at 8 weeks compared to baseline. Moreover, these effects were not influenced by the level of engagement with the app.

Importantly, the intervention showed a good level of acceptability among the participants. Most participants reported using the app at least once per week at both the 8- and 12-week time points. Participants also reported beginning to carry out their habits automatically at 8 and 12 weeks. Almost all respondents reported that they would like to see more similar services to this one provided by their local council. The findings suggest that older adults can achieve positive health outcomes through engaging with a health platform. The strong uptake observed may be attributed to several factors: the personalized nature of the app, which increases relevance and engagement; the recruitment strategy targeting individuals already motivated to improve their health; and the growing digital literacy among older adults, with 78% of those aged over 55 years owning smartphones in the United Kingdom [16].

The findings of this study are consistent with those of research indicating the potential for older adults to use mHealth interventions [17]. A systematic review identified that digital health interventions focused specifically on physical activity have been shown to improve balance and mobility and reduce falls in older adults [27]. Meanwhile, research suggests that older adults may engage with digital coaching programs at higher rates than their younger adult counterparts [28]. As such, these findings add to an emerging body of literature that suggests that older adults not only engage well with mHealth initiatives but can also experience positive health outcomes from doing so. A scoping review on digital health literacy in older adults indicates that many factors can influence it in this population [29]. This includes whether they already own a digital device and having more positive attitudes toward health knowledge and greater confidence in their ability to manage their health through their digital device [29].

To be eligible to take part in the study, participants were required to have access to a phone or tablet with internet access. Additionally, the fact that they would like to see similar services rolled out by their local council suggests that, among this sample at least, there is a clear interest in using digital devices to monitor one’s health. Moreover, our results add to the literature suggesting that personalized content in particular is beneficial [24,25]. Qualitative research on perceptions of mHealth interventions highlights that users particularly value personalized and tailored content [30]. Similarly, research in the domain of weight loss indicates that tailored content is more effective than content that is either a moderate or poor fit for the user [31]. Thus, Holly Health’s personalized features and content are beneficial for user experience, and future research should look into which elements of Holly Health offer the most benefit to its users.

The Holly Health app incorporates elements of behavior change theory, including self-determination theory, which posits that autonomy, competence, and relatedness are key to sustained behavior change. Features such as personalized goal setting and habit tracking may enhance users’ sense of autonomy and competence. Additionally, CBT and ACT elements may help users reframe negative thoughts and increase psychological flexibility, contributing to improved well-being.

The health survey measures showed significant improvements after the intervention. Scores in domains such as self-confidence, energy, mindfulness, and short- and long-term mindset were all higher after the intervention as compared to before. Thus, after engaging in the intervention, participants were more likely to endorse the importance of longer-term outcomes as opposed to motivation dwindling when short-term goals did not yield immediate rewards. A study on physical activity in older adults identified that participants were likely to set goals related to maintaining physical activity and preventing aging decline. Thus, the authors concluded that apps that encourage goal setting are likely to give rise to stronger internal motivation, which may increase the intervention’s effectiveness [32]. The findings of this study are in line with this, whereby participants were more likely to endorse a long-term mindset after engaging with the Holly Health app. This is important for ensuring that health behavior change is sustained. Participants also reported higher levels of mindfulness after engaging with the Holly Health intervention. Mindfulness refers to awareness of both one’s internal states and one’s surroundings [33]. Mindfulness stress reduction interventions have been shown to improve health-related behaviors [34] and improve quality of life and health behaviors among a sample of adults with hypertension [35]. As such, the significant increase in self-reported mindfulness in this study is a positive indicator that participants will be better able to engage in health behavior change after using the Holly Health app. In addition to statistical significance, effect size estimates (Cohen *d*) were calculated for all primary outcomes, which ranged from small to moderate. This suggests that, while the sample was underpowered, the observed changes could still be meaningful in a real-world context. Including effect sizes is especially important in feasibility studies because it helps show the potential impact of the intervention and informs sample size planning for future trials.

Participants’ health survey and FCQ scores at baseline significantly predicted the ONS-4 happiness scale scores at 12 weeks, demonstrating the link among physical health, food choices, and well-being. This is consistent with research finding positive relationships among physical exercise, physical health, and well-being among adults in midlife [36]. As research indicates that diet quality is poor among older adults in the European Union and United States [37], the findings of this study suggest that engaging with a digital health intervention can help improve physical health and nutrition and may even have positive benefits beyond physical health. Given the importance of nutrition for healthy aging, the findings present promising initial evidence as to the potential for Holly Health to be effective in promoting healthy aging.

This study has several limitations. First, it was a proof-of-concept study focused on the acceptability and preliminary efficacy of the Holly Health app. Consequently, the sample size was small (N=34) and not statistically powered, which limits the strength and generalizability of the findings. Additionally, the pretest-posttest design lacked a control group, introducing potential bias and restricting confidence in attributing observed effects solely to the intervention—making it difficult to rule out alternative explanations such as regression to the mean [38]. Second, the sample was predominantly women (28/36, 77.7%) and largely comprised White individuals (25/36, 69.4%). Gender imbalances are common in digital health research as women often show higher rates of participation and engagement—possibly due to greater interest in health apps and wellness interventions. However, this imbalance may still influence outcomes and limits generalizability. Future research should recruit gender-balanced and ethnically diverse samples to explore subgroup differences in engagement and efficacy. Moreover, the volunteer sampling method likely attracted adults already motivated to improve their health, introducing self-selection bias that may have inflated engagement and outcome measures [39]. Future studies should use more diverse recruitment strategies to capture a broader range of health motivation levels. The study also included only short-term follow-up at 8 and 12 weeks, limiting insights into the sustainability of behavior change and health outcomes. Future research should incorporate longer follow-up periods (eg, 6 to 12 months) to evaluate the maintenance of effects and long-term health outcomes. Finally, the study used some study-specific measures (eg, the health survey and retrospective questions) that have not been prevalidated. While these were tailored for exploratory purposes, their reliability and validity are unestablished, warranting caution in interpretation. Nevertheless, many items were derived from validated instruments such as the ONS-4, enhancing partial validity [40]. Future research should incorporate fully validated instruments to improve the comparability and robustness of the findings.

In summary, while this study demonstrated that the Holly Health app is effective in improving health outcomes in a sample of older adults and showed good acceptability, these findings should be interpreted with caution due to methodological limitations. Nonetheless, the results underscore the potential of personalized digital health interventions to empower older adults in self-managing their health. The strong engagement and reported improvements in self-confidence, energy, and mindfulness suggest that such tools can play a transformative role in promoting healthy aging. Future research should build on these findings through robust, controlled trials and validated measures to confirm efficacy and explore long-term impacts across diverse populations.

## Supplementary material

10.2196/62748 Multimedia Appendix 1 Holly Health survey.

10.2196/62748 Multimedia Appendix 2 Frequency of use and acceptability measures.

## Abbreviations

ACT: acceptance and commitment therapy
CBT: cognitive behavioral therapy
FCQ: Food Choice Questionnaire
mHealth: mobile health
NCD: noncommunicable disease
ONS-4: Office of National Statistics personal well-being questions
