# Supplementary material for: Evaluation of a Digital Intervention to Improve the Health Outcomes of Older Adults: Secondary Data Analysis
Source: J Particip Med. 2026 Jun 5;18:e62748. doi: 10.2196/62748 (PMC13240981; doi:10.2196/62748)
Supplement: Multimedia Appendix 1 [file jopm-v18-e62748-s001.docx]

Appendix A: Holly Health survey

1. **Physical activity**

How many hours a week do you do any type of moderate physical activity? (e.g. walking, swimming, running, cycling, yoga, etc)

- I don't do any exercise
- Less than 1 h/week
- 1-2 h/week
- 2-3 h/week
- 3+ h/week

1. **ONS-4: Scale from 0-10 (Personal wellbeing)**

Overall, how satisfied are you with your life nowadays?

- 0=Not at all
- 10= Completely

Overall, to what extent do you feel that the things you do in your life are worthwhile?

- 0=Not at all
- 10= Completely

Overall, how happy did you feel yesterday?

- 0=Not at all
- 10= Completely

On a scale where 0 is “not at all anxious” and 10 is “completely anxious”, overall, how anxious did you feel yesterday?

- 0=Not at all
- 10= Completely

1. **Self-confidence**

How would you rate your self-confidence?

- Very poor
- Poor
- Average
- Good
- Excellent

1. **Relationship with food:**

Which of these statements best describes how you relate to food?

- I tend to feel guilty when I eat certain foods, I try to restrict what I eat, and sometimes I end up overeating based on strong emotions
- I see some foods as 'good' or 'bad' and I tend to base my eating choices on different diet programmes
- I try to eat based on what I think is 'healthy' but it's not always possible
- I often eat what I fancy but sometimes I feel guilty if I eat more than usual
- Most of the time I base my eating choices on what I fancy and what makes me feel good

1. **Energy levels:**

How would you rate your average energy levels on any given day?

- I'm exhausted most of the time
- I'm pretty tired most days
- It varies quite a lot
- I often feel I have enough energy throughout the day
- My energy levels are great most of the time

1. **Mindfulness: *(Item 7 of MAAS scale, validated questionnaire)**

How much do you relate to the following statement:

***“It seems I am “running on automatic,” without much awareness of what I’m doing”***

- Almost always
- Very frequently
- Sometimes
- Very rarely
- Almost never

1. **Short vs long-term mindset:**

How much do you identify with the following statement when setting health and wellbeing goals:

***'I tend to set short-term goals with big gains in mind. I usually lose motivation if I don't see results quickly'***

- A lot. That's me pretty much every time I set new health and wellbeing goals
- Very much. Though sometimes I can keep going even if the results don't come as fast
- Somewhat. My motivation varies based on why I started the goal in the first place
- Not that much. Often I set health and wellbeing goals thinking of long-term outcomes
- Not at all. Most of the time when I set health and wellbeing goals, I focus on the small steps to get there and my motivation doesn't usually depend on the outcome

1. **Health mindset:**

When thinking about your health, which statement applies the most to you:

- "I don't think about my health at all"
- "I sometimes think about my health but I don't do anything to improve it"
- "I think about my health and I do a few things on and off to improve it"
- "I often think about my health and I do what I can to improve it"
- "Everyday I think about my health and I take actions to improve it"

1. **Self-kindness:  *(Item 12  of Self-compassion scale, validated questionnaire)**

Please choose the answer that best describes how you feel:

***“When I’m going through a very hard time, I give myself the caring and tenderness I need”***

- Almost always
- Very frequently
- Sometimes
- Very rarely
- Almost never
