# Supplementary material for: Evaluation of a Digital Intervention to Improve the Health Outcomes of Older Adults: Secondary Data Analysis
Source: J Particip Med. 2026 Jun 5;18:e62748. doi: 10.2196/62748 (PMC13240981; doi:10.2196/62748)
Supplement: Multimedia Appendix 2 [file jopm-v18-e62748-s002.docx]

Appendix B: frequency of use and acceptability measures

1. **Using Holly:**

Have you been using the Holly Health app (at least once a week) in the last (x)  weeks?

- Yes
- No

1. **(If yes) Automatic habits:**

Have you started to do any of your habits automatically? That is, without relying on the app to remind you?

- Yes
- No

1. **Usefulness:**

Overall, have you found Holly Health useful?

- Yes
- No

1. **Feedback:**

Do you have any specific feedback you'd like to share about the app?

- *Free text*

 In future, would you like to see more services like this provided by your local council?

- Yes
- No

Do you have any feedback about taking part in this study so far?

- *Free text*

**Additional retrospective questions for week 12 only:*

1. **Acceptability:**

In general, how appropriate did you find the Holly Health service as a tool for older adults to keep up with their healthy ageing goals?

- Not appropriate at all
- Not very appropriate
- Quite neutral
- Very appropriate
- Extremely appropriate

How likely are you to recommend Holly Health to other people with similar health and wellbeing goals as you?

- 1 (Not at all likely)
- 2
- 3
- 4
- 5 (Extremely likely)
